# A Case Report of Meningitis with Possible Coinfection by *Listeria monocytogenes* and *Mycobacterium tuberculosis* (Detected by Metagenomic Next‐Generation Sequencing) and Literature Review

**DOI:** 10.1155/crcc/9615951

**Published:** 2026-07-01

**Authors:** Hongyin Zhu, Pan Yang, Yunxia Tu, Xiaoyu Fu, Xian Yang, Na An

**Affiliations:** ^1^ Department of Internal Medicine Intensive Care Unit, First People′s Hospital of the Yunnan Province, Yunnan, China; ^2^ Emergency Medicine Department, Armed Police Yunnan Corps Hospital, Kunming, China

**Keywords:** coinfection, *Listeria* meningitis, sepsis, tuberculous meningitis

## Abstract

**Rationale:**

The study is aimed at exploring the complex clinical scenario of a patient with systemic lupus erythematosus who developed a rare coinfection with *Listeria monocytogenes* and *Mycobacterium tuberculosis*. The rationale is to highlight the diagnostic and therapeutic challenges in managing such a case, particularly in the context of immunosuppression and the need for effective antimicrobial therapy. This case underscores the importance of advanced diagnostic techniques like metagenomic next‐generation sequencing in identifying coinfections and the critical balance required in treating both infections while managing the underlying autoimmune condition.

**Patient Concerns:**

This case report presents a 58‐year‐old female patient who initially manifested thrombocytopenia and was diagnosed with SLE in an external hospital. After treatment, her condition did not improve. On the contrary, she developed a fever and a headache, and her disturbance of consciousness gradually worsened. The patient was admitted to our hospital with a suspected diagnosis of lupus encephalopathy and central nervous system infection.

**Diagnoses:**

MRI plain scan showed linear enhancement shadows in the right temporal pole and bilateral cerebellar hemisphere regions on the fluid‐attenuated inversion recovery three‐dimensional volumetric fluid‐attenuated inversion recovery contrast‐enhanced scan. Subsequently, NGS of the cerebrospinal fluid detected *L. monocytogenes* and *M. tuberculosis*, suggesting a possible mixed infectious meningitis caused by these two pathogens.

**Interventions:**

The patient underwent a comprehensive treatment regimen including anti*Listeria* and antituberculosis therapies. Unfortunately, this was followed by the development of liver failure and various other complications. In response, we administered interventions such as blood purification and liver support measures. Furthermore, we organized a multidisciplinary consultation to address the complex medical needs of the patient.

**Outcomes:**

Despite aggressive medical interventions, the patient′s condition deteriorated. She developed multiorgan failure, which significantly impacted her prognosis. The patient′s family elected to withdraw life‐sustaining treatment, and the patient passed away within 24 h after discharge.

**Lessons:**

This case underscores the importance of early and accurate diagnosis, particularly for immunocompromised patients with complex clinical presentations. Identifying mixed infections is crucial, and it also poses a significant challenge in selecting appropriate antimicrobial agents and conducting relevant tests.

## 1. Introduction

*Listeria monocytogenes* and *Mycobacterium tuberculosis* are two distinct pathogens known for causing severe infections in humans. Listeriosis, primarily caused by *L. monocytogenes*, is a foodborne illness that can lead to meningitis, encephalitis, and sepsis, particularly in immunocompromised individuals, pregnant women, neonates, and the elderly. On the other hand, tuberculosis caused by *M. tuberculosis* is a leading infectious disease worldwide, with the potential to affect various organs, including the central nervous system (CNS), where it manifests as tuberculous meningitis (TbM). TbM is a severe form of TB with high morbidity and mortality rates, especially when diagnosis is delayed. Mixed infections involving *L. monocytogenes* and *M. tuberculosis* are rare, and their cooccurrence in the CNS is even less common. This rarity poses significant challenges in terms of clinical recognition, diagnosis, and management. The clinical presentation of such mixed infections can be nonspecific, often overlapping with other CNS infections, thereby complicating the diagnostic process. Moreover, the treatment strategies for these pathogens differ, necessitating a precise diagnosis to initiate appropriate therapy promptly.

This case report presents a rare case of possible mixed meningitis caused by *L. monocytogenes* and *M. tuberculosis*. By detailing the clinical presentation, diagnostic approach, and management of this unique case, we aim to enhance the understanding of the complexities involved in diagnosing and treating mixed CNS infections. Furthermore, we seek to highlight the importance of maintaining a high index of suspicion for mixed infections in patients with CNS manifestations, particularly those with risk factors for both Listeria and tuberculosis.

## 2. Case Presentation

### 2.1. Ethics Approval and Consent to Participate

Informed written consent was obtained from the patient′s legally authorized representative (her husband) for the publication of this case report and accompanying images. This study was reviewed and approved by the local ethics committee of First People′s Hospital of Yunnan Province. The procedures were under the Helsinki Declaration of 1975, as revised in 2000.

### 2.2. Medical History

A patient who is 58 years old came to our hospital on March 14, 2025, with a four‐month history of thrombocytopenia. Her medical history includes: (A) Coronary artery disease (postcoronary stent placement) for over 1 year, managed with regular aspirin, clopidogrel, and rosuvastatin. (B) Hyperthyroidism for 12 years, treated with daily methimazole (1 tablet). (C) Type 2 diabetes mellitus for over 1 year, controlled with metformin. (D) Pulmonary tuberculosis 10 years prior was successfully treated with standard antituberculosis therapy for 1 year.

In April 2024, she developed pancytopenia (thrombocytopenia and leukopenia) with positive fecal occult blood. The suspected causative drug, methimazole, was discontinued immediately upon the discovery of pancytopenia. A follow‐up complete blood count 1 month later showed some improvement but persistent thrombocytopenia and leukopenia. Bone marrow biopsy suggested drug‐induced hematologic abnormalities. On March 7, 2025, she suddenly experienced severe bilateral lower extremity pain (right side worse than left) and sought care at a local hospital. She was preliminarily diagnosed with SLE and started on IVIG and high‐dose corticosteroid therapy. On March 10, she developed a fever and a headache without obvious triggers. By March 12, her condition worsened significantly, presenting with progressive altered mental status, urinary incontinence, and vomiting. She was transferred to our hospital and admitted to the Internal Medicine Intensive Care Unit following our department′s consultation.

### 2.3. Clinical Manifestations

• General condition: The patient had a body temperature of 38.3°C, blood pressure of 119/71 mmHg, heart rate of 157 bpm, and respiratory rate of 38 br/min.
• Neurological examination: The patient was in a shallow coma. Both pupils were equal in size and round, with absent light reflexes. Neck stiffness was present (measured as 2 fingerbreadths). Muscle tone in all four limbs was decreased, and there was no obvious response to painful stimuli. No pathological reflexes were elicited bilaterally.
• Other Findings: There were no obvious rashes on the body. Lung and abdominal examinations showed no significant abnormalities.

### 2.4. Auxiliary Examinations

#### 2.4.1. Key Laboratory Results

• Complete blood count: White blood cell count was 23.51 × 10^9^/L, with 22.89 × 10^9^/L neutrophils (97.3% of total), 1.4% lymphocytes, hemoglobin at 100 g/L, and platelet count of 26 × 10^9^/L. The whole‐blood C‐reactive protein was 111.65 mg/L.
• Blood biochemistry: Procalcitonin was 6.56 ng/mL. Potassium was 2.8 mmol/L, sodium 134.1 mmol/L, calcium 1.98 mmol/L, magnesium 0.74 mmol/L, inorganic phosphorus 0.42 mmol/L, glucose 8.25 mmol/L, total protein 86.3 g/L, albumin 28 g/L, alanine aminotransferase 15 U/L, aspartate aminotransferase 12 U/L, total bilirubin 8.5 *μ*mol/L, serum creatinine 45 *μ*mol/L, and blood urea nitrogen 4.1 mmol/L. The erythrocyte sedimentation rate was 120 mm/1 h, and interleukin‐6 was 128.3 pg/mL.
• Coagulation function: The activated partial thromboplastin time was 45.1 s, prothrombin time 19.1 s, fibrinogen 5.64 g/L, fibrinogen degradation products 50.12 *μ*g/mL, D‐dimer 10.22 *μ*g/mL, and prothrombin time activity 51%.
• Autoimmune antibody profile: Antinuclear antibodies were positive (homogeneous pattern, titer 1: 3200), antiENA was positive (with specific reactivity to antiSmith and antiSSA/Ro60), and double‐stranded DNA antibodies were weakly positive. Anticardiolipin antibodies were IgA 1.558 APLU/mL, IgG 11.964 GPL/mL, IgM 14.333 MPL/mL, and myeloperoxidase antibodies were weakly positive. Serum complement levels were significantly low: C3 was 0.44 g/L (reference 0.90–1.80 g/L) and C4 was 0.27 g/L (reference range 0.10–0.40 g/L). These findings, in conjunction with the clinical presentation of thrombocytopenia, met the diagnostic criteria for systemic lupus erythematosus. The electrocardiogram showed sinus tachycardia, with low‐flat and biphasic T waves in leads V2–V5.

#### 2.4.2. CSF Examination

Pressure is at 186 mmH_2_O. The appearance was yellow and slightly turbid. The total nucleated cell count was 183 × 10^6^/L, with 80% polymorphonuclear cells and 20% mononuclear cells. The protein level was 7.4 g/L, glucose was 4.4 mmol/L (while the simultaneous blood glucose was 19.7 mmol/L), and chloride was 145 mmol/L.

CSF microbiological examination (March 15): Gram staining of the smear was negative, no acid‐fast bacilli were found with acid‐fast staining, and TB‐DNA was negative.

#### 2.4.3. Imaging Examinations

**Figure 1:**
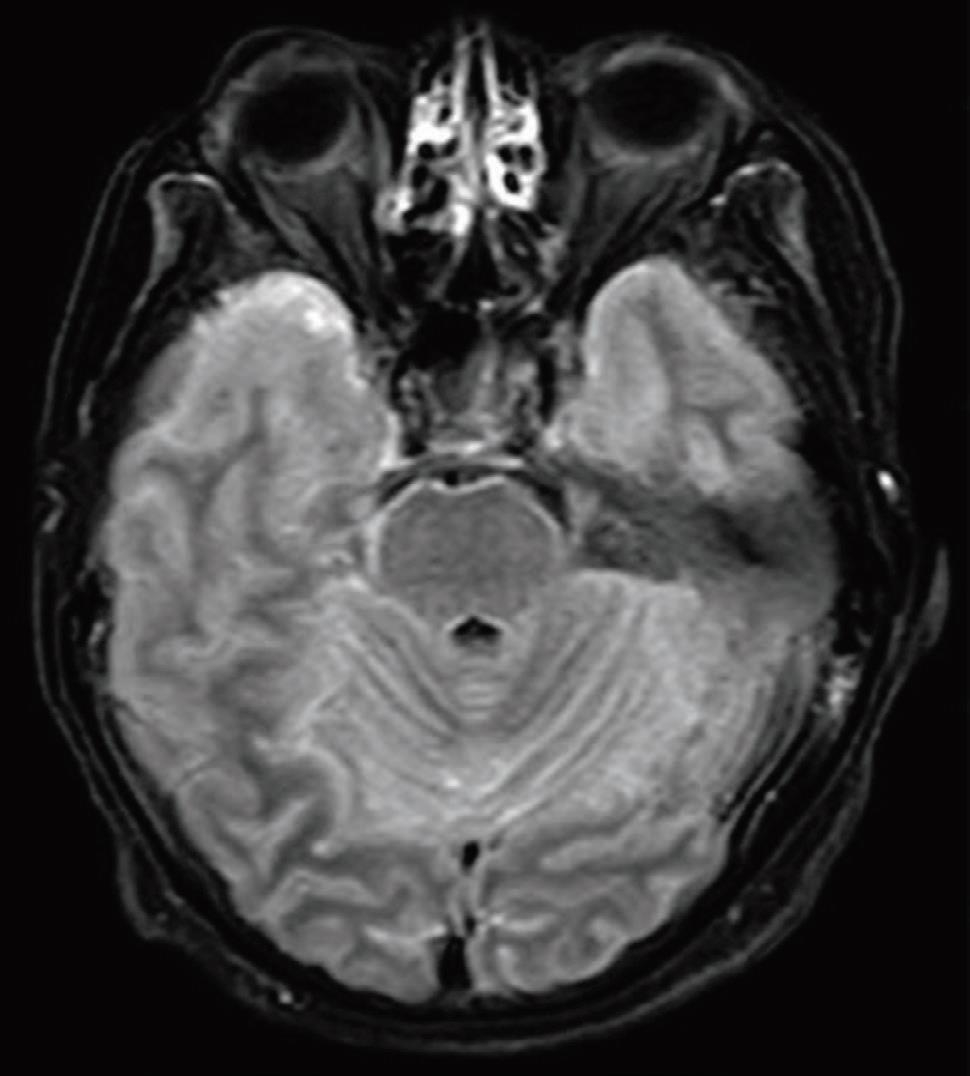
Linear enhancement in the right temporal pole on MRI.

**Figure 2:**
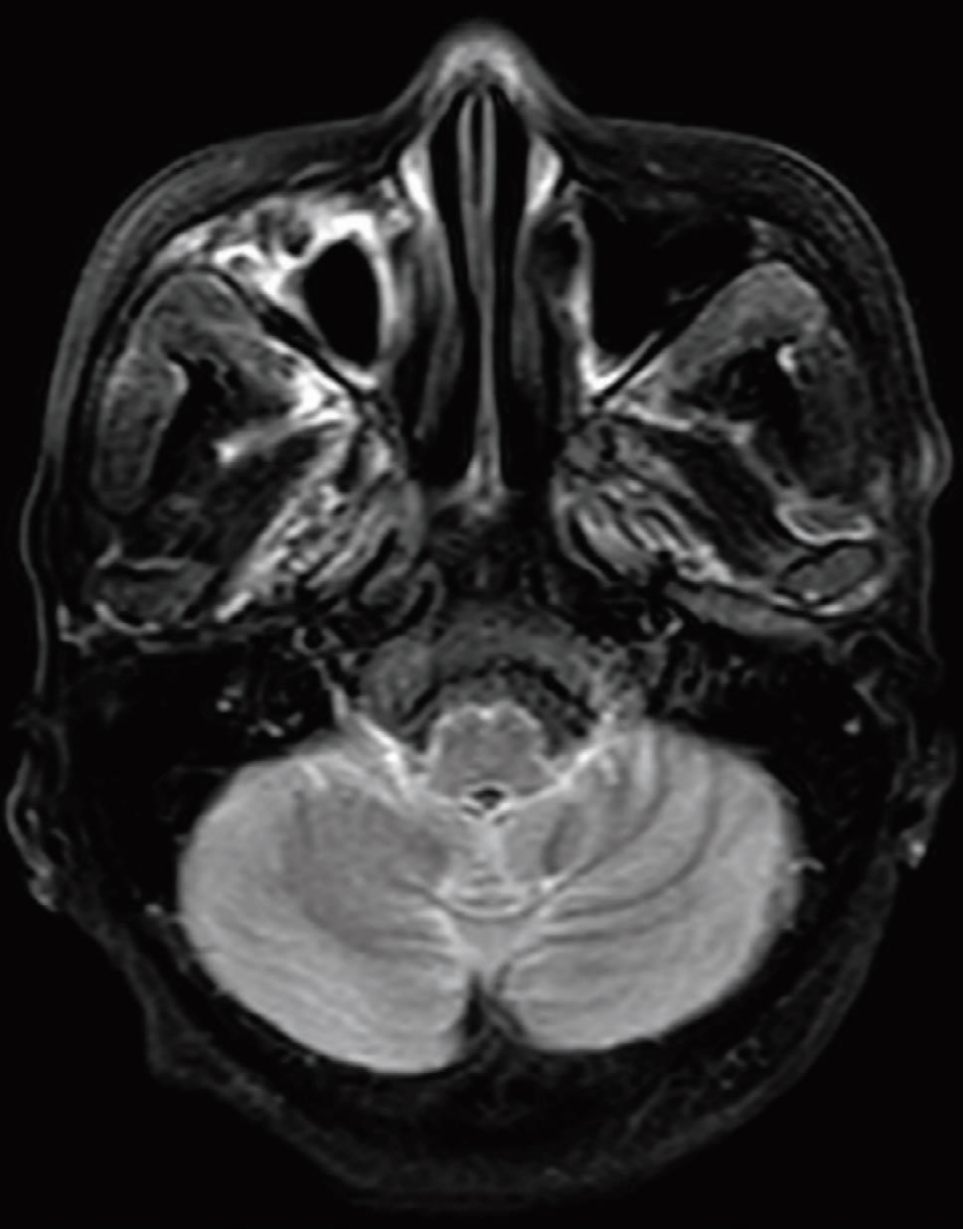
Linear enhancement in the cerebellar hemisphere on MRI.

CT scan showed suspicious patchy, slightly low‐density shadows in both cerebellar hemispheres. Noncontrast chest CT scan identified a bulla in the posterior segment of the right upper lobe and a solid nodule (approximately 7 × 6 mm) in the medial basal segment of the right lower lobe. These findings were considered likely sequelae, with no radiographic evidence of active tuberculous infiltration or cavitation. For magnetic resonance imaging (MRI) plain scan plus enhancement (Figures 1 and 2), there were patchy, slightly long T1 and T2 signal shadows in the right temporal pole. On the water‐suppression image, symmetrical strip‐like high‐signal shadows were seen in the leptomeningeal areas of both cerebellar hemispheres. On the water‐suppression three‐dimensional volume water‐suppression enhanced scan, linear enhancement shadows were suspected in the right temporal pole, bilateral temporal lobes, and bilateral cerebellar hemispheres. Meningoencephalitis was considered.

### 2.5. Diagnostic Process

Given the initial diagnosis of SLE made at the referring hospital, lupus encephalopathy was initially considered the most likely diagnosis. However, CNS infection could not be ruled out. Therefore, further CSF analysis was performed to determine the specific type of infection.

#### 2.5.1. Diagnostic Basis

On March 17, both CSF culture and blood culture yielded positive results for *L. monocytogenes*. On March 18, next‐generation sequencing (NGS) of CSF detected *L. monocytogenes* (with a sequence count of 426) and *M. tuberculosis* complex (with a sequence count of 1). Based on the microbiological evidence, the patient was diagnosed with listeriosis sepsis and *Listeria monocytogenes* meningitis (LM) .In addition, the NGS finding suggested possible concurrent TbM, although conventional tests (acid‐fast staining, TB‐DNA, Xpert) remained negative.

### 2.6. Treatment and Outcome

#### 2.6.1. Management of Systemic Lupus Erythematosus

The patient had received intravenous immunoglobulin (IVIG) pulse therapy at the referring hospital for suspected SLE. Following rheumatology consultation at our hospital on March 15, intravenous methylprednisolone (80 mg intravenously daily) was initiated. Further immunosuppressive agents were deferred due to the concurrent life‐threatening mixed infection. However, upon confirmation of *M. tuberculosis* coinfection by CSF NGS on March 18, the methylprednisolone was discontinued after a three‐day course. This decision was made through multidisciplinary consultation, prioritizing the management of the active, life‐threatening mycobacterial infection over the immunosuppressive therapy for SLE, due to the high risk of exacerbating tuberculosis dissemination.

#### 2.6.2. Antimicrobial Therapy

• Initial empiric therapy: Meropenem 1 g intravenously every 8 h was initiated upon admission to cover potential pathogens.
• *L. monocytogenes*‐targeted therapy: Upon confirmation of *L. monocytogenes* infection on March 17, treatment was escalated to ampicillin 2 g intravenously every 6 h in combination with gentamicin 80 mg every 8 hs.
• *M. tuberculosis*‐targeted therapy: Following the diagnosis of *M. tuberculosis* complex infection on March 18, antituberculosis therapy was initiated with isoniazid 300 mg orally once daily, and rifampicin 450 mg once daily. Pyrazinamide 1.5 g orally once daily, and ethambutol 750 mg orally once daily.
• Supportive care measures: Osmotic diuresis with mannitol to reduce intracranial pressure; parenteral mecobalamin for neuroprotection; norepinephrine infusion for hemodynamic support; blood glucose control regimen; hepatoprotective therapy; blood product transfusion as needed.

#### 2.6.3. Treatment Course and Outcome

• Day 1: The patient presented with coma, high fever, and respiratory failure, necessitating endotracheal intubation and mechanical ventilation. CSF analysis revealed 80% neutrophils and a protein level of 7434 mg/L.
• Day 3: Diagnosis of *L. monocytogenes* infection was confirmed through CSF and blood cultures, prompting the initiation of treatment with ampicillin and gentamicin.
• Day 5: NGS identified both *L. monocytogenes* and *M. tuberculosis* complex, leading to the addition of quadruple antituberculosis therapy.
• Day 6: A repeat lumbar puncture was performed, and Xpert testing was completed. CSF analysis showed 1488 nucleated cells, 90% neutrophils, a protein level of 169,044 mg/L (markedly elevated), and chloride levels at 98 mmol/L.
• Day 7: Marked elevation of liver enzymes (AST 818 U/L) indicated impending hepatic failure, necessitating discontinuation of antituberculosis medications and transition to second‐line therapy with levofloxacin.
• Day 8: Rapid increases in liver enzymes and bilirubin levels signified worsening hepatic failure. Despite negative Xpert results, multidisciplinary consultation raised concerns for secondary hemophagocytic syndrome, with ongoing clinical deterioration and plans for advanced liver support therapy. However, the family opted to withdraw treatment. The patient remained comatose throughout the entire ICU course.
• Day 9: The patient was discharged while still comatose at the family′s request and passed away at home on the same day, as confirmed by follow‐up.

## 3. Discussion

LM and TbM are both severe CNS infections with high mortality and morbidity rates. They share similarities in high‐risk populations and clinical manifestations but differ significantly in transmission routes, diagnostic methods, and treatment. *L. monocytogenes* is a Gram‐positive facultative intracellular bacterium primarily transmitted through ready‐to‐eat foods, dairy products, undercooked or inadequately heated meats, and processed meats [1]. In contrast, TbM is predominantly caused by hematogenous dissemination of *M. tuberculosis*. According to the World Health Organization (WHO), the global incidence of listeriosis ranges from 0.1 to 10 cases per million people annually, posing significant public health concerns despite its relatively low prevalence [2]. TbM represents the most severe form of tuberculosis, with at least 100,000 cases reported globally each year [3]. Untreated TbM is fatal, and even with treatment, the prognosis remains poor, often accompanied by neurological sequelae. High‐risk groups for listeriosis include neonates, the elderly, pregnant women, immunocompromised individuals, and those with malignancies or diabetes [4], although TbM is more common in patients with compromised immunity or chronic debilitating diseases. Clinical manifestations of LM include fever, headache, nausea, vomiting, and often atypical meningeal signs; TbM presents similarly with headache, vomiting, and meningeal signs [5, 6]. These similarities and nonspecific symptoms pose diagnostic challenges, particularly in immunocompromised patients, where accurate differentiation between the two diseases is crucial. Diagnosis relies on CSF analysis and imaging, with TbM typically showing lymphocytic pleocytosis, elevated protein levels, and reduced glucose in the CSF, whereas LM′s CSF profile is less characteristic. Comparing these features (Table 1) aids in diagnosis. These distinctions and overlaps present significant challenges for clinicians in diagnosing and treating these conditions, especially in immunocompromised patients, where precise differentiation is essential.

**Table 1:** Laboratory characteristics comparison.

CSF parameters	Typical presentation of LM	Typical presentation of TbM	Characteristics in this case
Cell classification	Predominantly neutrophils	Predominantly lymphocytes	80% neutrophils
Glucose	Normal or mildly decreased	Significantly decreased	4.4 mmol/L (concurrent blood glucose 19.7 mmol/L)
Protein	Moderately elevated	Significantly elevated	7434 mg/L → 169,044 mg/L
Sodium	Often accompanied by hyponatremia	May be accompanied by hyponatremia	Peak value 193 mmol/L

Imaging studies play a pivotal role in the diagnosis of CNS infections, aiding clinicians in distinguishing between various types of infections. The characteristic MRI findings for LM include hyperintense signals on T2‐weighted imaging (T2WI) and fluid‐attenuated inversion recovery (FLAIR) sequences in the brainstem and cerebellum, potentially accompanied by ring‐enhancing lesions [7, 8]. Additionally, CT scans may reveal hypodense lesions in the brainstem and pons, possibly involving the cerebellum, or manifest as diffuse rhombencephalic swelling with occlusion of the cerebellopontine cistern, followed by the development of microabscesses or larger abscesses within the brainstem or cerebellum. In contrast, the characteristic imaging features of TbM differ, as described by Subedi et al. [9] who noted that MRI typically shows a combination of basal meningeal enhancement, tuberculomas, infarcts, or hydrocephalus. Although these findings can provide diagnostic clues, they are not definitive for diagnosis. Nonetheless, the imaging manifestations of LM and TbM often lack specific, typical features, posing significant challenges for diagnosis. Clinically, the codiagnosis of both infections is even more complex, necessitating a comprehensive analysis that integrates clinical symptoms, laboratory tests, and imaging findings.

Microbiological results are crucial for confirming CNS infections, necessitating thorough CSF smear and culture analyses. However, the sensitivity of traditional acid‐fast bacilli smears for *M. tuberculosis* is only 10%–15% [10], whereas cultural sensitivity is higher (approximately 50%–60%), but it requires an extended period (2–6 weeks, depending on the medium) [11]. This delay can impede timely treatment, potentially leading to rapid disease progression and patient mortality. Consequently, empirical treatment and rapid diagnostic methods are of paramount importance. Hannan et al. [12] demonstrated that PCR testing of CSF is an effective method for diagnosing TbM. This molecular assay detects the genetic material of *M. tuberculosis* and determines its resistance to rifampicin, with a sensitivity of 31%–100% and specificity of 66%–100% [10]. For *L. monocytogenes*, PCR testing exhibits high sensitivity and specificity, but it requires prior primer design, limiting its detection range and necessitating multiple or distributed testing in cases of mixed infections. NGS complements PCR by offering broader coverage for detecting unidentified infections. A large multinational study [13] estimated that LM and TbM account for 1.0% and 5.8% of all CNS infection cases, respectively. NGS is particularly suitable for diagnosing CNS infections of unknown origin, especially when traditional tests are negative [14, 15]. This method offers higher sensitivity and can simultaneously detect multiple coinfecting pathogens. Combining these findings with the present case, an analysis and summary of the diagnostic efficacy for LM and TbM are presented in Table 2.

**Table 2:** Analysis of diagnostic technology efficacy.

Method	Listeria detection rate	Tuberculosis detection rate	Application in this case
Gram staining	30%–40%	10%–15%	Negative
Culture	3–5 days	2–6 weeks	Listeria positive (3 days)
Xpert MTB/RIF	Not applicable	50%–60%	Negative
NGS	81.27%[16]	98%[17]	Double positive (72 h)

It is important to acknowledge a key limitation of metagenomic NGS in this case: the detection of only one sequence read for *M. tuberculosis* complex. Although NGS is highly sensitive for identifying rare or unexpected pathogens, the clinical interpretation of a very low sequence count—especially in the absence of corroborating positive results from conventional tests such as acid‐fast staining, TB‐DNA PCR, and Xpert MTB/RIF—requires caution. In this patient, the single positive NGS read for *M. tuberculosis* was considered a possible indicator of true infection rather than environmental contamination or background noise, given her history of prior pulmonary tuberculosis and profoundly immunocompromised state. Nevertheless, the diagnosis of TbM in this case remains probable rather than definitive. The NGS finding should be regarded as a complementary clue that guided antituberculosis therapy, not as standalone proof of active infection.

Both LM and TbM are life‐threatening emergencies. Their clinical presentations are diverse yet share certain similarities. However, they are caused by different pathogens and require different treatment regimens. Delayed treatment often leads to poor prognoses, with high mortality and disability rates [18, 19]. Misdiagnosis and missed diagnosis are highly likely, making early diagnosis and timely treatment crucial for improving outcomes. Cases of coinfection with LM and TbM are relatively rare in clinical practice. A retrospective study [20] revealed that TbM patients have a milder inflammatory response but more severe CNS complications. LM patients often have an immunosuppressed state, whereas TbM patients do not. These findings can help clinicians more accurately distinguish between TbM and LM, enabling the formulation of more appropriate treatment plans [20].

In this case, the patient presented with a complex immunocompromised state. The thrombocytopenia was initially suspected to be drug‐induced, considering her long‐term methimazole use and the bone marrow biopsy findings suggestive of drug‐induced abnormalities. However, the cytopenias persisted despite methimazole discontinuation. In conjunction with the subsequent development of high‐titer autoantibodies and hypocomplementemia; the thrombocytopenia was ultimately more indicative of active SLE. Subsequently, the administration of high‐dose corticosteroids for SLE management, combined with the underlying immune dysregulation of SLE itself, created a profound immunosuppressed state. This state likely predisposed her to the subsequent rare coinfection with *L. monocytogenes* and *M. tuberculosis*. There may be a hypothesis of sequential infection: the patient′s immunosuppressed state reactivated the latent *M. tuberculosis* from 10 years ago. The reinfected *M. tuberculosis* produced cytokines that could disrupt the blood‐brain barrier and increase its permeability, which is a key virulence feature of *M tuberculosis* [3], providing favorable conditions for the dissemination of tuberculosis and the spread of *L. monocytogenes*. However, except for the CNS, all the tuberculosis test results of the patient were negative. The author is more inclined to believe that the infection of *L. monocytogenes* exacerbated the patient′s immunosuppressed state, and under this state, either reactivated the latent *M. tuberculosis* or caused a new tuberculosis infection. Therefore, in high‐risk populations such as the elderly and those with chronic diseases, the possibility of mixed infection with both or multiple pathogens should be considered. According to the literature [21], immunosuppressive therapy is an independent risk factor for LM, and *L. monocytogenes* has a relatively high incidence in the elderly population. Therefore, in the elderly and immunocompromised populations, prevention and monitoring of *L. monocytogenes* disease should be strengthened.

In terms of treatment, distinct therapeutic regimens are required for LM and TbM, and delayed treatment is associated with poor prognosis. A combined anti‐infective approach targeting both pathogens is crucial, alongside active management of underlying conditions (such as diabetes) to improve outcomes. Regarding antibiotic selection, *L. monocytogenes* is inherently resistant to cephalosporins. Currently, ampicillin‐class penicillins combined with aminoglycosides like gentamicin are the first‐line treatments in clinical practice [22], with a treatment duration of 4–8 weeks. Compared to benzylpenicillin and ampicillin, patients treated with meropenem have a higher mortality rate [23]. For the treatment of TbM, the WHO recommends that both adults and children initially receive a four‐drug regimen (rifampicin, isoniazid, pyrazinamide, and ethambutol) for 2 months, followed by 7–10 months of treatment with rifampicin and isoniazid [24, 25]. However, the incidence of liver damage caused by antituberculosis drugs cannot be overlooked. During treatment, liver function tolerance must be closely monitored, and adjustments to the antituberculosis medications should be made promptly. In this case, after the patient developed liver damage, quinolones (levofloxacin was used in this case) were employed as a second‐line alternative for antituberculosis treatment [26].

In this case, after the patient was diagnosed with meningitis caused by a mixed infection of *L. monocytogenes* and *M. tuberculosis*, acute liver failure occurred during treatment, but the specific cause remains unclear. It is difficult to determine whether the liver is damaged by *L. monocytogenes* or the side effects of antituberculosis drugs. A review article by Scholing et al. [27]. reported that in previous adult disseminated listeriosis, reports of liver involvement were relatively rare, but animal models have shown that the portal vein system is widely involved in the early stages of invasive‐monocytosis caused by *L. monocytogenes*. Therefore, the author suspects that listeriosis presenting as severe liver dysfunction may not be rare. In addition, *Listeria* infection can easily induce severe sepsis. A systematic review and meta‐analysis [28] showed that the overall incidence of sepsis secondary to *L. monocytogenes* infection is approximately 31%–61%. Sepsis can lead to multiple organ dysfunction, and liver injury or liver failure is one of its common and serious complications. However, the liver damage caused by antituberculosis drugs cannot be excluded. The incidence of liver damage caused by the combined use of rifampin (15%) and isoniazid (20%) is as high as 35% [17]. Unfortunately, even after adjusting the antituberculosis drugs and receiving active liver‐protecting treatment, the patient′s liver function has not been reversed. Since the specific cause of liver damage is still unknown, in terms of the treatment plan for this case, at the treatment level, whether to prioritize covering *L. monocytogenes*, first control the sepsis, and then gradually start antituberculosis treatment, or start the treatment of both *L. monocytogenes* and tuberculosis simultaneously, poses a huge challenge to clinical treatment.

## 4. Conclusion

LM and TbM coinfection is relatively rare, characterized by a dual pathological feature of “rapid progression (*Listeria*) + chronic damage (tuberculosis),” which warrants attention from clinicians. For infectious diseases that initially present with subtle gastrointestinal disturbances followed by neurological manifestations, especially in the elderly and immunocompromised patients, heightened vigilance for neurolisteriosis is essential. Early diagnosis and combined antimicrobial therapy are pivotal for improving outcomes. NGS should be employed as a frontline diagnostic tool for CNS infections in immunosuppressed patients [14]. Additionally, meticulous management of underlying conditions is imperative to mitigate the occurrence of opportunistic infections.

**Nomenclature**
CNS: central nervous system
CSF: cerebrospinal fluid
IVIG: intravenous immunoglobulin
LM: *Listeria monocytogenes* meningitis
NGS: next‐generation sequencing
PCR: polymerase chain reaction
SLE: systemic lupus erythematosus
TbM: tuberculous meningitis

## Author Contributions

Data curation: Yunxia Tu, Xian Yang, and Hongyin Zhu. Investigation: Xiaoyu Fu. Writing—original draft: Hongyin Zhu. Writing—review and editing: Hongyin Zhu and Pan Yang.

## Funding

No funding was received for this manuscript.

## Disclosure

This case report was approved by the Ethics Committee of First People′s Hospital of the Yunnan Province.

## Consent

Written informed consent was secured from the patient and his family.

## Conflicts of Interest

The authors declare no conflicts of interest.

## Data Availability

The data that support the findings of this study are available from the corresponding author upon reasonable request.
